# 3D whole-heart isotropic sub-millimeter resolution coronary magnetic resonance angiography with non-rigid motion-compensated PROST

**DOI:** 10.1186/s12968-020-00611-5

**Published:** 2020-04-16

**Authors:** Aurélien Bustin, Imran Rashid, Gastao Cruz, Reza Hajhosseiny, Teresa Correia, Radhouene Neji, Ronak Rajani, Tevfik F. Ismail, René M. Botnar, Claudia Prieto

**Affiliations:** 1 Department of Biomedical Engineering, School of Biomedical Engineering and Imaging Sciences, King’s College London, St Thomas’ Hospital, 3rd Floor, Lambeth Wing, London, SE1 7EH UK; 2 National Heart and Lung Institute, Imperial College London, London, UK; 3 MR Research Collaborations, Siemens Healthcare Limited, Frimley, UK; 4 Department of Cardiology, Guy’s & St Thomas’ Hospitals, London, UK; 5 Escuela de Ingeniería, Pontificia Universidad Católica de Chile, Santiago, Chile

**Keywords:** Accelerated imaging, Coronary MR angiography, Isotropic sub-millimeter resolution. PROST reconstruction non-rigid motion compensation

## Abstract

**Background:**

To enable free-breathing whole-heart sub-millimeter resolution coronary magnetic resonance angiography (CMRA) in a clinically feasible scan time by combining low-rank patch-based undersampled reconstruction (3D-PROST) with a highly accelerated non-rigid motion correction framework.

**Methods:**

Non-rigid motion corrected CMRA combined with 2D image-based navigators has been previously proposed to enable 100% respiratory scan efficiency in modestly undersampled acquisitions. Achieving sub-millimeter isotropic resolution with such techniques still requires prohibitively long acquisition times. We propose to combine 3D-PROST reconstruction with a highly accelerated non-rigid motion correction framework to achieve sub-millimeter resolution CMRA in less than 10 min. Ten healthy subjects and eight patients with suspected coronary artery disease underwent 4–5-fold accelerated free-breathing whole-heart CMRA with 0.9 mm^3^ isotropic resolution. Vessel sharpness, vessel length and image quality obtained with the proposed non-rigid (NR) PROST approach were compared against translational correction only (TC-PROST) and a previously proposed NR motion-compensated technique (non-rigid SENSE) in healthy subjects. For the patient study, image quality scoring and visual comparison with coronary computed tomography angiography (CCTA) were performed.

**Results:**

Average scan times [min:s] were 6:01 ± 0:59 (healthy subjects) and 8:29 ± 1:41 (patients). In healthy subjects, vessel sharpness of the left anterior descending (LAD) and right (RCA) coronary arteries were improved with the proposed non-rigid PROST (LAD: 51.2 ± 8.8%, RCA: 61.2 ± 9.1%) in comparison to TC-PROST (LAD: 43.8 ± 5.1%, *P* = 0.051, RCA: 54.3 ± 8.3%, *P* = 0.218) and non-rigid SENSE (LAD: 46.1 ± 5.8%, *P* = 0.223, RCA: 56.7 ± 9.6%, *P* = 0.50), although differences were not statistically significant. The average visual image quality score was significantly higher for NR-PROST (LAD: 3.2 ± 0.6, RCA: 3.3 ± 0.7) compared with TC-PROST (LAD: 2.1 ± 0.6, *P* = 0.018, RCA: 2.0 ± 0.7, *P* = 0.014) and non-rigid SENSE (LAD: 2.3 ± 0.5, *P* = 0.008, RCA: 2.5 ± 0.7, *P* = 0.016). In patients, the proposed approach showed good delineation of the coronaries, in agreement with CCTA, with image quality scores and vessel sharpness similar to that of healthy subjects.

**Conclusions:**

We demonstrate the feasibility of combining high undersampling factors with non-rigid motion-compensated reconstruction to obtain high-quality sub-millimeter isotropic CMRA images in ~ 8 min. Validation in a larger cohort of patients with coronary artery disease is now warranted.

## Background

Three-dimensional (3D) whole-heart coronary magnetic resonance angiography (CMRA) has shown potential for the non-invasive diagnosis of coronary artery disease (CAD) without radiation exposure [1, 2]. However, CMRA still requires long scan times to achieve whole-heart coverage and sub-millimeter isotropic spatial resolution. Moreover, scan time is highly unpredictable due to the need of dealing with respiratory motion. Therefore, enabling free-breathing whole-heart sub-millimeter isotropic resolution CMRA in a short and predictable scan time by combining advanced motion correction and reconstruction techniques with a highly undersampled acquisition would be a major advance.

Conventional CMRA relies on a 1D diaphragmatic navigator [3, 4] to minimize respiratory motion by acquiring data only within a pre-defined small gating window and using a fixed correction factor of 0.6 [5]. This approach however requires a model to relate the motion of the diaphragm with the motion of the heart [6, 7] and a broad deviation in optimal correction factors can be found between subjects [8]. Furthermore, it leads to low respiratory scan efficiency and unpredictable scan times, since rejected data needs to be re-acquired. Self-navigation has been proposed to estimate the 1D foot-head [9–11] and 3D [12] translational motion of the heart from the data itself and has been used to enable 100% respiratory scan efficiency. However, since this approach does not account for the complex motion of the heart, residual motion artefacts may affect image quality. Moreover, due to the projection nature of the 1D self-navigated signal, the presence of static tissue in the field-of-view (FOV) may affect the accuracy of motion estimation. Translational respiratory motion correction using low-resolution two-dimensional (2D) or 3D image-based navigators (iNAVs) has recently been proposed for CMRA [9, 10, 13–17]. With these approaches, the heart can be spatially isolated from surrounding static tissues and 2D/3D translational respiratory motion can be estimated and corrected in a beat-to-beat fashion. To correct for remaining non-rigid (NR) motion, and thus to further improve CMRA image quality, respiratory binning techniques [18–21] have been employed in concert with iNAV-based translational motion correction and motion-compensated reconstruction [22, 23]. Most of these approaches have been successfully applied to fully sampled or mildly undersampled acquisitions with spatial resolution in the order of 1.2 mm [22–25], however further modifications are needed to extend these techniques to the higher undersampling factors needed to enable sub-millimeter Cartesian CMRA.

Several approaches have been proposed to accelerate CMRA data acquisition including undersampling reconstruction techniques and the previously mentioned motion correction approaches with 100% respiratory scan efficiency. While parallel imaging reconstruction techniques are conventionally used to reduce the acquisition time in CMRA [26, 27], these techniques are still limited to undersampling factors of 2–3 times. Further acceleration can be achieved using compressed sensing [28] at the expense of potential unnatural image appearance (so called staircasing artefacts) for very high undersampling factors. More recently, undersampled 2D and 3D patch-based reconstruction techniques, that exploit the inherent redundancies of the complex anatomy of the coronary arteries in an effective and efficient low-rank framework, have been proposed for CMRA [28, 29]. Those techniques have been successfully combined with diaphragmatic navigator [30] and 2D iNAV-based translational motion-correction [29] to achieve whole-heart sub-millimeter isotropic resolution CMRA with high image quality in less than 10 min. However, remaining NR respiratory motion may degrade image quality, especially affecting the visualization of distal coronary segments [22].

In this study, we aim to achieve sub-millimeter resolution 3D whole-heart CMRA in a predictable scan time of less than 10 min and accounting for the complex non-rigid respiratory motion of the heart. This is achieved by combining a 3D patch-based low-rank reconstruction (PROST) [29] with the matrix formalism for NR motion correction [22, 31]. This so-called NR-PROST framework is investigated in healthy subjects and a small cohort of patients with suspected coronary artery disease.

## Methods

In vivo acquisitions were performed on a 1.5 T CMR scanner (Magnetom Aera, Siemens Healthineers, Erlangen, Germany) with a dedicated 32-channel spine coil and an 18-channel body coil. Written informed consent was obtained from all subjects before undergoing CMRA scans and the study was approved by the National Research Ethics Committee. Image reconstructions and coronary analysis were performed offline on a workstation with a 16-core Dual Intel Xeon Processor (2.3 GHz, 256 GB RAM).

### Accelerated CMRA acquisition

**Fig. 1:**
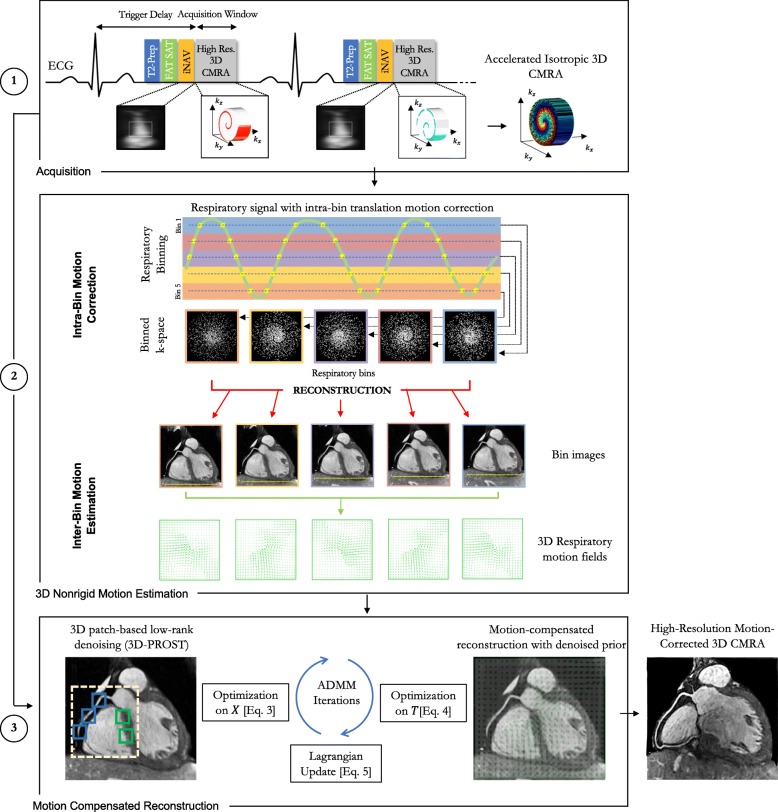
Schematic overview of the proposed accelerated free-breathing 3D coronary magnetic resonance angiography (CMRA) acquisition with sub-millimeter isotropic resolution, 100% scan efficiency and non-rigid motion-compensated patch-0based low-rank reconstruction (PROST) reconstruction. **1**) CMRA acquisition is performed with an undersampled 3D variable density spiral-like Cartesian trajectory with golden angle between spiral-like interleaves (VD-CASPR), preceded by 2D image navigators (iNAV) to allow for 100% scan efficiency and beat-to-beat translational respiratory-induced motion correction of the heart. **2**) Foot-head respiratory signal is estimated from the 2D iNAVs and used to assign the acquired data into 5 respiratory bins and translation-corrected respiratory bins. Subsequent reconstruction of each bin is performed using soft-gated SENSE and 3D non-rigid motion fields are then estimated from the 5 reconstructed datasets. **3**) The final 3D whole-heart motion-corrected CMRA image is obtained using the proposed 3D patch-based (PROST) non-rigid motion-compensated reconstruction. Abbreviations: CMRA, coronary magnetic resonance angiography; PROST, patch-based undersampled reconstruction; ADMM, alternating direction method of multipliers

An accelerated free-breathing 3D whole-heart electrocardiogram (ECG)-triggered, balanced steady-state free precession (bSSFP) sequence with a 3D variable density (VD) spiral-like Cartesian trajectory (VD-CASPR) with golden-angle step was employed as previously proposed [29] (Fig. 1a and Additional file 1). A low-resolution 2D iNAV preceded each spiral-like interleave to allow for 100% scan efficiency, predictable scan time and 2D translational motion correction of the heart on a beat-to-beat basis. The 2D iNAVs were obtained by spatially encoding the startup profiles of the bSSFP sequence [9]. A SPIR (spectral presaturation with inversion recovery) fat saturation pulse with a constant flip angle (FA) of 130° was used to improve coronary depiction and minimize fat-related aliasing artefacts. An adiabatic T2-preparation pulse [32, 33] was played at each heartbeat in order to enhance the contrast between blood and cardiac muscle and avoid the use of extracellular contrast agents.

### Motion correction and image reconstruction

The proposed framework for sub-millimeter free-breathing CMRA consists of 3 different stages that are described in more details hereafter: i) iNAVs-based 2D translational beat-to-beat respiratory motion estimation and correction; ii) respiratory binning and bin-to-bin non-rigid motion estimation; and iii) non-rigid motion-corrected 3D PROST reconstruction. The first two stages have been previously proposed for fully sampled non-rigid motion-corrected SENSE reconstruction [22]. A schematic overview of the proposed framework is shown in Fig. 1.

#### Beat-to-beat 2D translational motion correction

Beat-to-beat 2D translational motion correction was performed as previously proposed in [22, 23]. Briefly, foot-head (FH) and right-left (RL) translational respiratory motion of the heart was extracted from the iNAVs using a template-matching algorithm with normalized cross-correlation as similarity measure [9]. The reference template was manually selected during scan planning on a region encompassing the subject’s heart. The FH respiratory signal was used to sort the acquired data into 5 respiratory states or bins. Intra-bin 2D translational motion correction was performed by correcting the data for each bin to the same respiratory position (taken as the bin center). This correction was implemented by modulating the k-space data with a linear phase shift according to the previously estimated respiratory motion [22].

#### Bin-to-bin non-rigid motion estimation

Since the acquired 3D CMRA data is accelerated (5-fold), the resulting binned k-spaces are highly accelerated (~ 25-fold). Soft-gating sensitivity encoding reconstruction [22] was employed to reconstruct each respiratory bin. Bin-to-bin 3D non-rigid motion estimation was subsequently performed using spline-based free-form deformation [34], considering the end-expiration bin as the reference image (Fig. 1).

#### 3D patch-based non-rigid motion-compensated reconstruction (NR-PROST)

The estimated 3D non-rigid motion fields are then directly incorporated into a general matrix description reconstruction framework [31, 35]. As opposed to previous CMRA studies where 3D data was acquired fully sampled [22] or with mild acceleration factors [23], our proposed sub-millimeter CMRA framework exploits higher undersampling factors (5-fold) to reach sub 10-min acquisition times for sub-millimeter spatial resolution, leading to noise amplification associated with the ill-posed reconstruction problem.

3D-PROST has recently been proposed to highly accelerate sub-millimeter CMRA imaging with translational motion correction only [29]. 3D-PROST reconstruction exploits the inherent redundancies of the complex 3D anatomy of the coronary arteries on a local (i.e., within a patch) and non-local (i.e., between similar patches within a neighborhood) basis, through an efficient iterative low-rank decomposition and thresholding. The proposed NR-PROST framework combines 3D-PROST with the matrix formalism for non-rigid motion correction, and can be formulated as the following unconstrained optimization:

1 $$ {\mathcal{L}}_{NR- PROST}\left(X,\mathcal{T},Y\right):= \underset{X,\mathcal{T},Y}{\mathrm{argmin}}\ {\left\Vert EX-K\right\Vert}_F^2+\lambda {\left\Vert \mathcal{T}\right\Vert}_{\ast }+\frac{\mu }{2}{\left\Vert \mathcal{T}-X-\frac{Y}{\mu}\right\Vert}_F^2 $$

2 $$ E=\sum \limits_{b=1}^{N_{bins}}{A}_b FS{U}_b $$

where *X* is the non-rigid motion-corrected 3D CMRA volume, *K* is the 2D translation corrected k-space data, *E* is the encoding operator composed of: *A*_*b*_ the sampling matrix for bin *b*, *F* the 3D Fourier transform, *S* the coil sensitivities, *U*_*b*_ the estimated 3D non-rigid motion fields for bin *b* and *N*_*bins*_ the number of respiratory bins. ‖.‖_*F*_ and ‖.‖_∗_ denote the Frobenius and nuclear norms respectively, $\mathcal{T}$ is a self-similarity matrix built on a patch scale (see *Optimization 2* below) and *Y* is the Lagrangian parameter. The positive parameter *μ* controls the tradeoff between the regularization and the fidelity of the reconstructed motion-corrected 3D CMRA image with regard to the acquired data, whereas *λ* controls the denoising level.

Similar to [29, 36], Eq. (1) can be efficiently solved by operator-splitting via alternating direction method of multipliers (ADMM), which divides the optimization process into three iterative simpler sub-problems:

#### Optimization on X: non-rigid motion-compensated reconstruction

The first optimization is a motion-compensated reconstruction that incorporates the dealiased/denoised 3D volume $\mathcal{T}$ obtained at the end of optimization 2 as prior information:

3 $$ {\mathcal{L}}_{NR}(X):= \underset{X}{\mathrm{argmin}}\ {\left\Vert EX-K\right\Vert}_F^2+\frac{\mu }{2}{\left\Vert \mathcal{T}-X-\frac{Y}{\mu}\right\Vert}_F^2 $$

The above optimization reduces to a regularized non-rigid SENSE reconstruction where an a priori guess (${X}^{\ast }=\mathcal{T}-\frac{Y}{\mu }$) of the desired solution is incorporated into a Tikhonov-type regularization. This equation can be efficiently solved using the conjugate gradient (CG) algorithm.

#### Optimization on $\mathcal{T}$: 3D patch-based denoising

The second optimization is a 3D patch-based dealiasing/denoising applied to the previously reconstructed motion-compensated 3D CMRA volume *X*. This problem is formulated on a patch scale as:

4 $$ {\mathcal{L}}_{PROST}\left(\mathcal{T}\right):= \underset{\mathcal{T}}{\mathrm{argmin}\ }\frac{2\lambda }{\mu}\sum \limits_p{\left\Vert {\mathcal{T}}_p\right\Vert}_{\ast }+\sum \limits_p{\left\Vert {\mathcal{T}}_p-{P}_p(X)-\frac{Y_p}{\mu}\right\Vert}_F^2 $$

The details to solve the above equation can be found in [29]. In brief, the operator *P*_*p*_(.) extracts similar 3D patches from a reference patch centered at pixel *p* and builds a self-similarity 2D matrix ${\mathcal{T}}_p$ made by vectorizing and concatenating each similar 3D patch. The high degree of redundancy in ${\mathcal{T}}_p$ is exploited through low-rank matrix decomposition and singular value thresholding [37]. The truncation of the singular values, controlled by the thresholding parameter *λ*, acts as a dealiasing/denoising filter by providing a low-rank approximation of the original self-similarity 2D matrix. This procedure is repeated in a sliding window manner for all the pixels in the image and the final 3D volume $\mathcal{T}$ is obtained after aggregation. The aggregation step consists on positioning each denoised patch back onto the image and performing averaging if patches have been selected multiple times. A video describing the 3D patch-based dealiasing/denoising step for CMRA images is provided in Additional file 2.


**Additional file 2** Schematic video of the *Optimization 2* involved in the proposed non-rigid motion-compensated NR-PROST reconstruction. In this step, 3D dealiasing/denoising is performed using 3D block-matching, which groups similar 3D patches in the 3D volume, followed by a low-rank decomposition and thresholding of each group. This step is repeated for all the voxels, and the final 3D volume is obtained by aggregating the dealiased/denoised groups. This volume is then used as a prior in the motion-compensated reconstruction (*Optimization 1*) in order to regularize the reconstruction process.


##### Lagrangian multiplier Y update

Finally, the Lagrangian multiplier *Y* is updated by integration of the residual between the reconstructed motion-compensated 3D CMRA image and the dealiased/denoised 3D prior:

5 $$ Y=Y+X-\mathcal{T} $$

#### Healthy subject acquisitions

Ten healthy subjects (31 ± 7 years, range 25–52 years; 4 females) underwent CMRA imaging using the proposed approach. Relevant scan parameters included: 3D bSSFP sequence, coronal plane, echo time = 1.6 ms, repetition time = 3.7 ms, bandwidth per pixel = 890 Hz, FOV = 320 × 320 × 86–115 mm^3^, FA = 90°, T2-preparation duration = 40 ms, SPIR fat-saturation FA = 130°, 0.9 mm^3^ acquired isotropic resolution (reconstructed resolution 0.6 mm^3^), acceleration factor of 5x, and 14 linear ramp-up pulses for iNAV. Free-breathing cardiac cine imaging was performed prior to CMRA to determine subject-dependent mid-diastolic trigger delay and acquisition window length.

#### Patient acquisitions

The feasibility and preliminary clinical performance of the proposed framework was assessed in 8 patients with suspected coronary artery disease (51 ± 7 years; 3 females). Acquisition parameters were the same as described above for the healthy subject cohort. The acceleration factor was adjusted (between 4x and 5x) in order to achieve sub 10-min acquisition time irrespective of the number of slices required to cover the whole-heart. Baseline clinical characteristics of the patients are shown in Table 1. Prior to the CMRA acquisitions, the eight patients underwent a clinically-indicated coronary computed tomography angiography (CCTA) with third generation dual source CT 192 × 2-sections (SOMATOM Force, Siemens Healthineers) which included sublingual glyceryl trinitrate and intravenous beta-blocker (metoprolol). CCTA images were reconstructed to 0.6 mm^3^ isotropic resolution.

**Table 1:** Baseline characteristics and sequence-relevant parameters for the patient study (*N* = 8)

Patient Characteristics	Patient 1	Patient 2	Patient 3	Patient 4	Patient 5	Patient 6	Patient 7	Patient 8
Age (yo)	52	60	54	50	53	53	35	54
Gender (M/F)	F	F	M	M	F	M	M	M
Body mass index (kg/m^2^)^a^	28.2	21.8	30.4	26.9	32	26	24	33
Time interval between CCTA/CMRA (days)	4	150	10	10	32	33	11	4
Average R-R interval – CMRA (ms)	1041 ± 19	928 ± 31	947 ± 21	997 ± 58	908 ± 30	1170 ± 43	1052 ± 51	1058 ± 73
Average R-R interval – CCTA (ms)	950	1083	1395	937	882	1463	952	1277
SNR-relevant sequence parameters								
Acceleration factor	5	4	4	4	4	5	5	5
Resolution (mm^3^)	0.9	0.9	0.9	0.9	0.9	0.9	0.9	0.9
Imaging time (min:s)	8:37	4:53	9:27	7:42	10:07	9:48	7:58	9:18
No. of k-space lines per heartbeat	22	32	27	27	22	22	22	22
Acquisition window (ms)	80	113	97	95	77	75	80	80
No. of slices	114	96	137	114	106	106	106	114

^a^Calculated as the weight in kilograms divided by the square of the height in meters; *CCTA* coronary computed tomography angiography; *CMRA* coronary magnetic resonance angiography

### Motion-compensated CMRA reconstruction

Reconstruction parameters for the proposed NR-PROST approach were empirically optimized on several datasets (not reported here) and were maintained for all reconstructions. Similar patches were searched in a 3D window of size 40^3^ voxels. The size of the 3D patches, controlling the degree of structural information within each patch, was set to 5^3^ voxels. The number of selected similar patches was set to 20. The performance of NR-PROST was evaluated by comparing reconstruction quality (not reported here) with several thresholding parameters *λ* and regularization parameters *μ*. The chosen parameters were *λ* = 0.1 and *μ* = 0.3. A good tradeoff between image quality and computational speed was obtained for 5 outer ADMM iterations, each performing 7 CG iterations for optimization 1. A gain in reconstruction speed can be further obtained by adding a small patch offset when searching for similar patches [38]. An offset of 4 voxels in the three dimensions showed small to no impact on the reconstructed image quality, while the gain in computational speed was substantial. We therefore set this offset to 4 in all reconstructions. The acquired data was also reconstructed with: 1) 3D-PROST and 2D beat-to-beat translational motion correction only [29], referred to as TC-PROST; and 2) non-rigid motion-compensated SENSE (termed NR-SENSE) reconstruction [22], which consists of solving Eq. [3] without prior (i.e., *μ* = 0). Reconstruction parameters were set to match those of the proposed NR-PROST technique.

All reconstructions were implemented in MATLAB (v7.1, The MathWorks, Natick, Massachusetts, USA). Optimization 2 of NR-PROST was implemented in C using multi-core CPUs and interfacing with MATLAB. Coil sensitivity maps were estimated from the k-space center using the eigenvalue-based approach ESPIRiT [39].

### Image analysis

To evaluate the performance of the proposed approach in healthy subjects, each reconstructed CMRA image was reformatted along the left anterior descending (LAD) and right (RCA) coronary arteries using a dedicated software [40]. Vessel sharpness (first 4 cm and full vessel length) and maximum visible vessel length of both coronary arteries were computed. A vessel sharpness of 100% indicates an abrupt change in normalized signal intensity (0 to 1) whereas a sharpness value of 0% implies the absence of an edge. Additionally, two blinded experienced cardiologists (I.R. and T.F.I. with 4 and 10 years of experience, respectively, SCCT accreditation and SCMR level III certification) reviewed in a randomized order the reconstructed images (i.e., NR-PROST, NR-SENSE and TC-PROST) for each subject and evaluated the quality of the CMRA images. For each subject, and both LAD and RCA, the reconstruction techniques were given a rank from worst (score 1) to best (score 3). Additionally, the quality of both LAD and RCA delineations was assessed using a 4-point scoring system where 1: indicates uninterpretable images, 2: poor image quality (blurred edges, noise and residual artefacts), 3: acceptable image quality (LAD/RCA adequately visualized, only mildly blurred edges), 4: excellent image quality with sharply defined coronary borders. In those cases where the two experts disagreed, the images were reviewed again together, and a consensus was made.

Statistical analyses using repeated-measures one-way analysis of variance (ANOVA) with Bonferroni correction for post-hoc comparisons (vessel sharpness and vessel length) and Wilcoxon signed rank test (experts scoring and ranking) were performed to examine whether the values obtained with the three reconstruction techniques were statistically different. All statistical analyses were performed using MATLAB (v7.1, The MathWorks), and statistical significances were defined as two-tailed values of *P* < 0.05.

For the patient study, the reconstructed 3D CMRA and CCTA images were transferred to a workstation and 3D curved planar reformations were conducted (Horos software, v3.3.4). Vessel sharpness was measured for the first 4 cm of the proximal segments of the LAD and the RCA in the 8 patients for the proposed CMRA approach. Image quality of the reconstructed CMRA images using NR-PROST and breath-held contrast-enhanced CCTA was assessed by the same two blinded experts who assessed the healthy subject data, for both LAD and RCA. The same 4-point scoring system, as described above for the healthy subjects, was used.

## Results

Free-breathing whole-heart CMRA was successfully completed in all healthy subjects and patients. Average total reconstruction times were ~ 2 min (TC-PROST), ~ 20 min (NR-SENSE) and ~ 50 min for NR-PROST.

### Healthy subject study

**Fig. 2:**
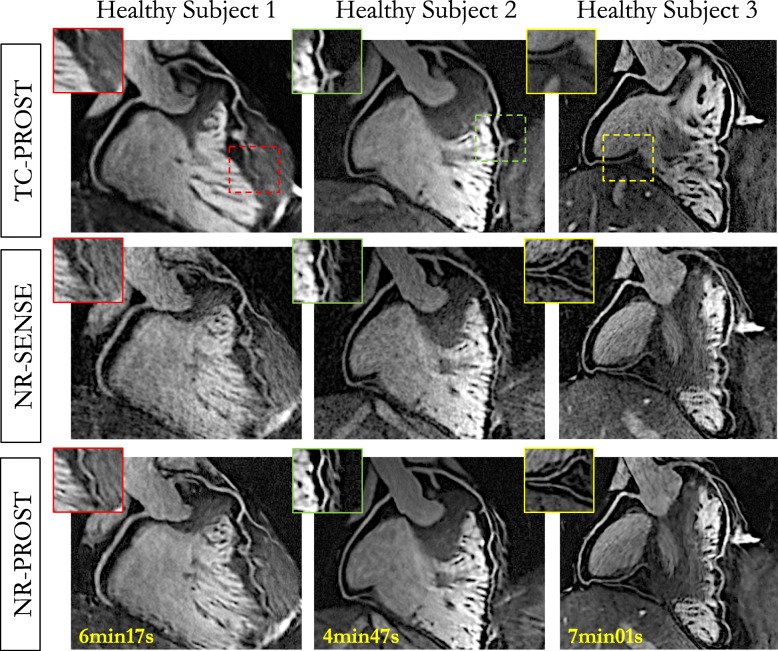
CMRA images reformatted along the left anterior descending (LAD) and right coronary artery (RCA) for 3 representative healthy subjects. Acquisitions were performed with 0.9 mm^3^ isotropic resolution and 100% respiratory scan efficiency. 3D CMRA images were obtained using the proposed non-rigid motion-compensated PROST reconstruction (NR-PROST, bottom row), non-rigid motion-compensated SENSE (NR-SENSE, middle row) and translational motion correction only with PROST reconstruction (TC-PROST, top row). Scan times are expressed as min:s

**Fig. 3:**
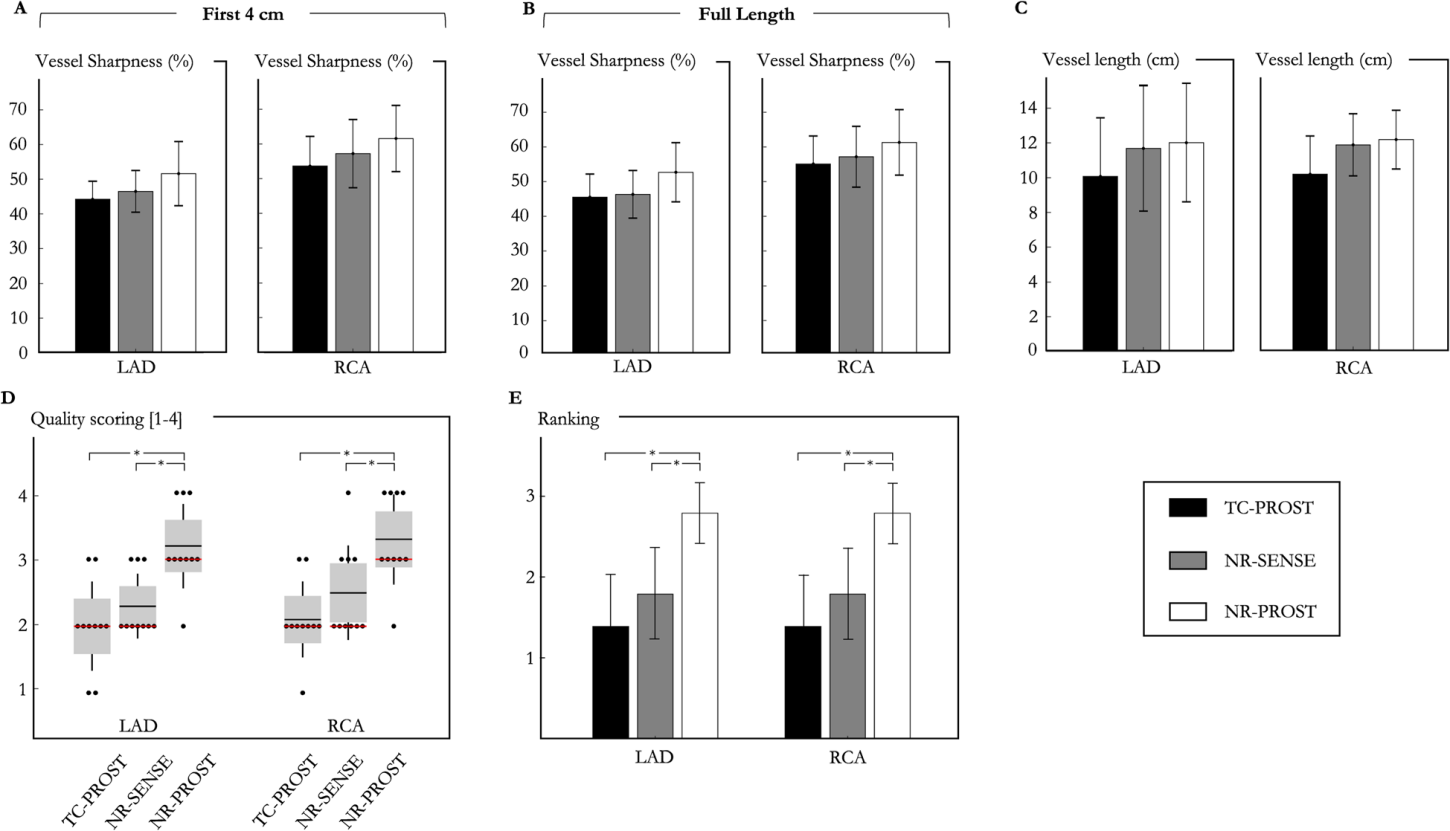
Quantitative and qualitative coronary metrics for sub-millimeter isotropic CMRA images for 10 healthy subjects for translational motion correction only (TC-PROST, black), non-rigid motion-compensated SENSE reconstruction (NR-SENSE, grey) and the proposed non-rigid motion-compensated PROST (NR-PROST, white). Vessel sharpness results are shown for the first 4 cm (**a**) and the full length (**b**), and coronary vessel length are depicted in (**c**). Qualitative visual scores of the LAD and RCA (**d**) and reconstruction rankings (**e**) are shown for the three reconstruction techniques. Results are expressed as mean ± standard deviation. Differences with statistical significance are identified by *P < 0.05. The dotted red lines depict the median values. Abbreviations: LAD, left anterior descending artery; RCA, right coronary artery

The average total scan time for the healthy subject study was 6:01 ± 0:59 [min:s] (range 4:19–7:32), with 100% respiratory scan efficiency. The mean heart rate (in beats per minute [bpm]) and data acquisition window were 58 ± 8 bpm (range 42–68 bpm) and 106 ± 13 ms (range 78–125 ms), respectively. Mean subject-dependent mid-diastolic trigger delay was 697 ms (range 508–963 ms). CMRA images reformatted along the LAD and RCA for 3 representative healthy subjects are shown in Fig. 2. While TC-PROST reconstruction provides high image quality with good visualization of the coronary arteries, correcting for remaining non-rigid motion with NR-SENSE enables improved coronary visualization, particularly of the distal segments, at the cost of some noise amplification. NR-PROST further improves image quality and the delineation of both LAD and RCA (Fig. 2). Clear depiction of the coronary arteries, including distal segments and major branches, can be observed with a comparable image quality between subjects (Additional file 3). Vessel sharpness, defined by the maximum signal intensity change at the vessel edge, was higher with NR-PROST (first 4 cm, LAD: 51.2 ± 8.8%, RCA: 61.2 ± 9.1%) than NR-SENSE (first 4 cm, LAD: 46.1 ± 5.8%, *P* = 0.223, RCA: 56.7 ± 9.6%, *P* = 0.50) and TC-PROST (first 4 cm, LAD: 43.8 ± 5.1%, *P* = 0.051, RCA: 54.3 ± 8.3%, *P* = 0.218) but showed no statistical significance, as observed in Fig. 3a-b. The same observation applies for the visible vessel length (Fig. 3c) where NR-PROST shows improved depiction of the coronary vessel (full vessel length, LAD: 11.8 ± 3.3 cm, RCA: 12.2 ± 1.6 cm) compared with NR-SENSE (LAD: 11.5 ± 3.5 cm, *P* = 0.980, RCA: 11.8 ± 1.7 cm, *P* = 0.91) and TC-PROST (LAD: 9.7 ± 3.5 cm, *P* = 0.336, RCA: 10.3 ± 2.1 cm, *P* = 0.71), although differences were not statistically significant. The average visual score (Fig. 3d), as given by the two experts, was significantly higher for NR-PROST (LAD: 3.2 ± 0.6, RCA: 3.3 ± 0.7) compared with NR-SENSE (LAD: 2.3 ± 0.5, *P* = 0.008, RCA: 2.5 ± 0.7, *P* = 0.016) and TC-PROST (LAD: 2.1 ± 0.6, *P* = 0.018, RCA: 2.0 ± 0.7, *P* = 0.014), ranking NR-PROST as the best reconstruction technique in 80% of the cases for both LAD and RCA visualizations (Fig. 3e).

### Patient study

The average total scan time for the patient study was 8:29 ± 1:41 [min:s] (range 4:53–10:07), with 100% respiratory scan efficiency. The mean heart rate and data acquisition window were 60 ± 5 bpm (range 51–66 bpm) and 87 ± 13 ms (range 75–113 ms), respectively. Five out of the 8 patients presented with CAD. The mean time interval between the CCTA and the CMRA acquisitions was 32 days (range 4 to 150 days).

**Fig. 4:**
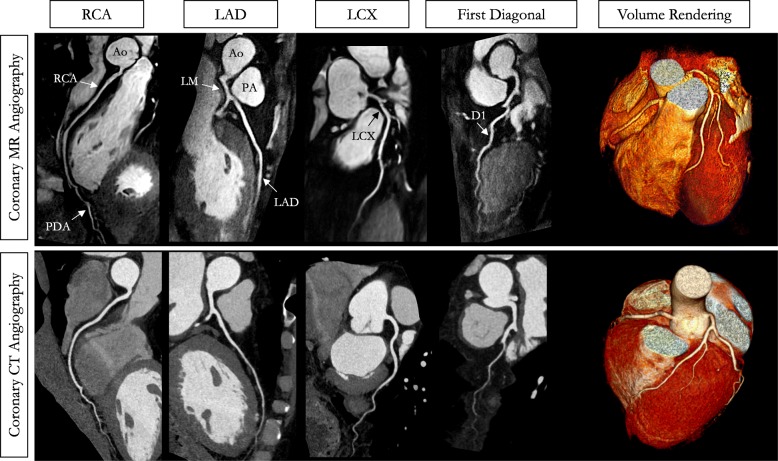
Non-contrast whole-heart sub-millimeter isotropic CMRA images of a 53-year-old male patient with normal coronary arteries (patient 6). Accelerated free-breathing CMRA images reconstructed with non-rigid motion-compensated PROST are shown in the top row, revealing the RCA, LAD, left circumflex (LCX) and first diagonal territories. The 3D CMRA dataset was acquired in 9min48s (heart rate of 51 bpm). The corresponding reformatted images obtained with contrast enhanced CCTA are shown in the bottom row. Left anterior three-dimensional volume-rendered images for both modalities are shown in the right-hand column, showing good delineation of the left and right coronary systems. Abbreviations: CMRA, coronary magnetic resonance angiography; LAD, left anterior descending artery; RCA, right coronary artery; LCX, left circumflex artery; PDA, posterior descending artery; PA, pulmonary artery; Ao, aorta

**Fig. 5:**
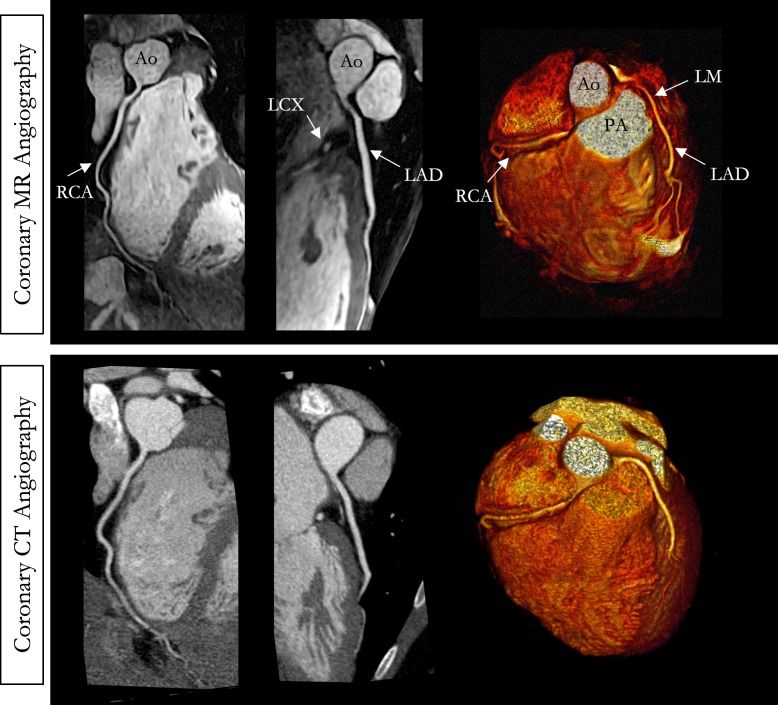
Non-contrast whole-heart sub-millimeter isotropic CMRA images of a 35-year-old male patient with normal coronary arteries (patient 7). Accelerated free-breathing coronary MR angiography (CMRA) images reconstructed with non-rigid motion-compensated PROST are shown in the top row, revealing the LAD and RCA coronary arteries. The 3D CMRA dataset was acquired in 7 min 58 s (heart rate of 57 bpm). The corresponding reformatted images obtained with contrast-enhanced CT coronary angiography are shown in the bottom row. Three-dimensional volume-rendered images for both imaging modalities are shown in the right-hand column. Abbreviations: LAD, left anterior descending artery; RCA, right coronary artery; LCX, left circumflex artery; LM, left main; Ao, aorta; PA, pulmonary artery

A representative CMRA dataset from a 53-year-old healthy male patient and a 35-year-old male patient with normal coronary arteries are shown in Fig. 4 and Fig. 5, respectively, with the corresponding CCTA images. CMRA with 0.9 mm^3^ isotropic resolution combined with NR-PROST reconstruction allows for good visualization of the LAD, RCA and left circumflex (LCX) territories from the proximal to the distal segments with good image quality and visual image sharpness. Additional non-contrast whole-heart sub-millimeter isotropic 3D CMRA images in three patients are shown in Additional file 4.

**Fig. 6:**
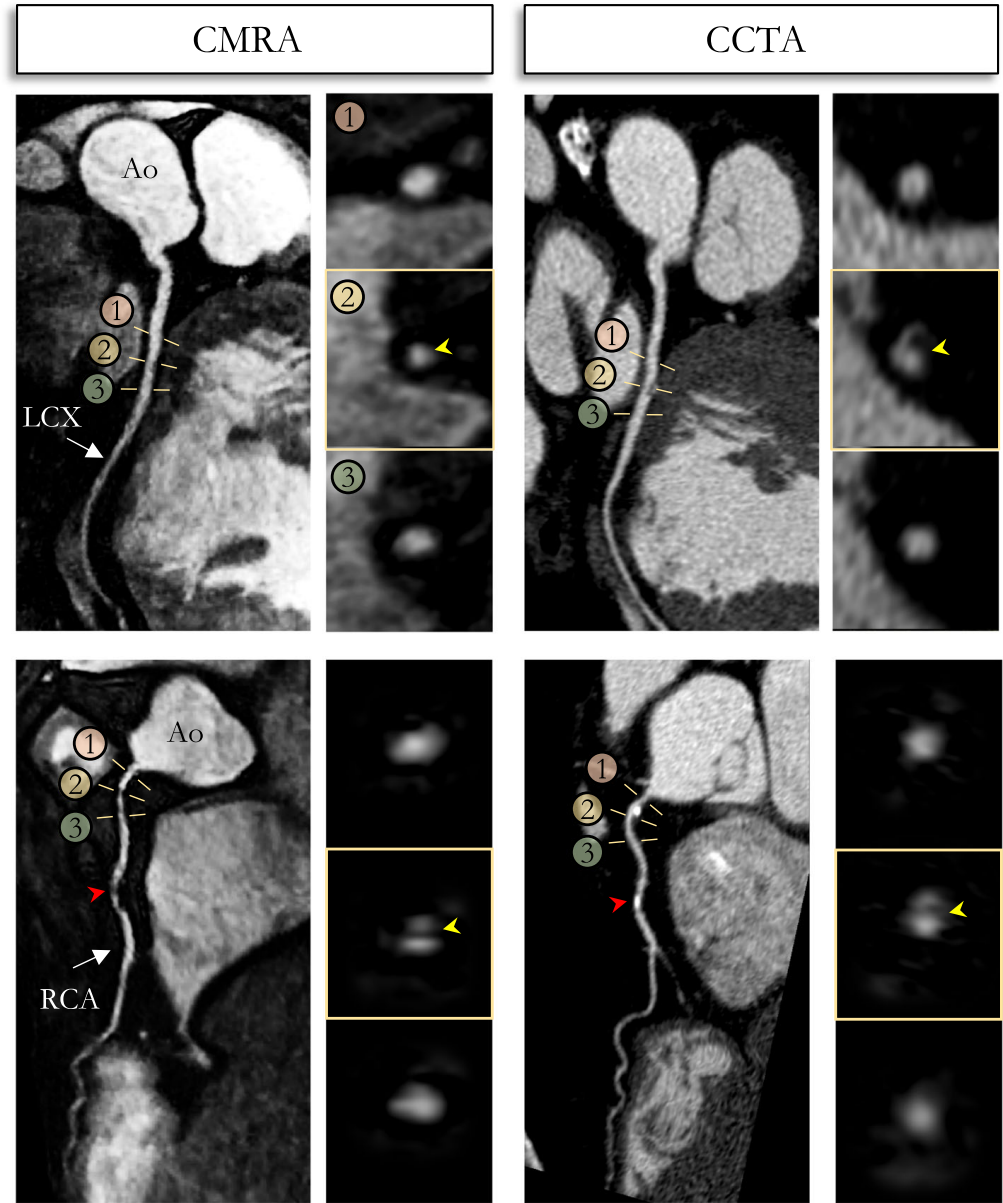
Reformatted non-contrast whole-heart sub-millimeter isotropic CMRA (left) and CCTA (right) images along the LCX (top) and RCA (bottom) are shown for a 54-year-old male patient (patient 8). The CMRA dataset was acquired in 9 min with 100% scan efficiency (heart rate of 57 bpm). The CCTA images demonstrate mild (25–49%) disease with a calcified plaque within the proximal RCA and severe disease (70–90%) with a partially calcified plaque in the mid-segment of RCA (red arrows), and minimal (0–24%) disease with calcified plaque in the mid-segment of the LCX. Luminal narrowing is seen on the cross-sectional views at the sites of coronary plaque on the CMRA images (yellow arrows). Abbreviations: CMRA, coronary magnetic resonance angiography; LAD, left anterior descending artery; RCA, right coronary artery; LCX, left circumflex artery; Ao, aorta

**Fig. 7:**
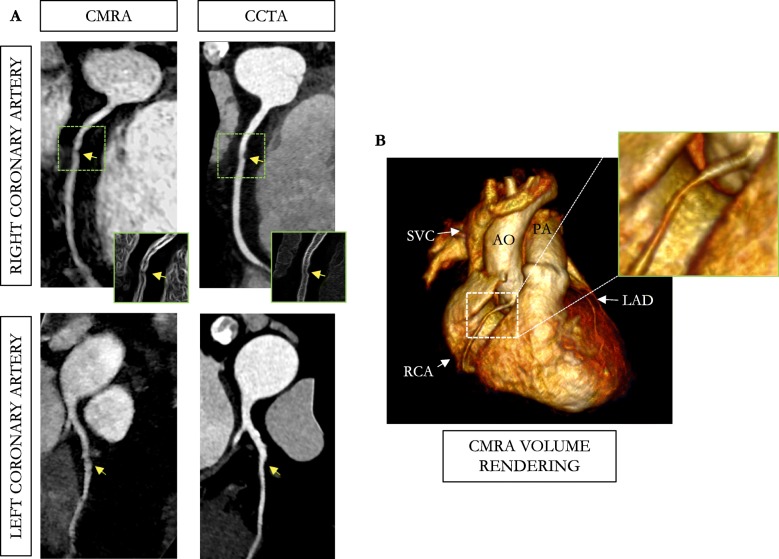
CMRA (0.9 mm^3^ resolution) in a 52-year-old female patient (patient 1) with coronary artery disease. The CMRA data was acquired in 8 min 37 s with 100% scan efficiency at an average heart rate of 58 bpm. **a** The reformatted CMRA images (left-hand column) disclose a stenosis (arrow) in the mid RCA that matches the lesion observed on the CCTA (right-hand column). A significant narrowing was observed on both modalities with a 50% diameter reduction, compared with normal reference proximal diameter. The same patient showed calcified coronary plaque in the mid LAD (calcium mass: 2.88 mg, Agatston score: 16.1) leading to luminal narrowing of the mid and distal segments of the coronary artery observed on the CMRA and CCTA (arrow). **b** Volume rendered image of the 3D CMRA dataset is shown. Abbreviations: CMRA, coronary magnetic resonance angiography; CCTA, coronary computed tomography angiography; LAD, left anterior descending artery; RCA, right coronary artery; SVC, superior vena cava; AO, aorta; PA, pulmonary artery

Reformatted non-contrast enhanced CMRA images obtained in a 54-year-old male patient with left and RCA disease are shown in Fig. 6, with the corresponding CCTA images. The CCTA images demonstrate mild (25–49%) disease with calcified plaque within the proximal RCA segment, a severe stenosis (70–90%) with partially calcified plaque within the mid-segment of the RCA, and a minimal (0–24%) disease with calcified plaque in the mid LCX segment. Luminal narrowing of the arteries can be seen on the cross-sectional views at the sites of coronary plaque on the CMRA images (Fig. 6, yellow arrows). Another CMRA dataset obtained in a 52-year-old female patient with left and RCA disease is shown in Fig. 7. The CMRA reformat (Fig. 7a) and corresponding volume rendering (Fig. 7b) reveal a plaque visible in the proximal RCA which is confirmed on the CCTA image. A significant stenosis of the mid segment of the LAD is also observed leading to luminal narrowing of the coronary artery on the CMRA and CCTA images.

**Fig. 8:**
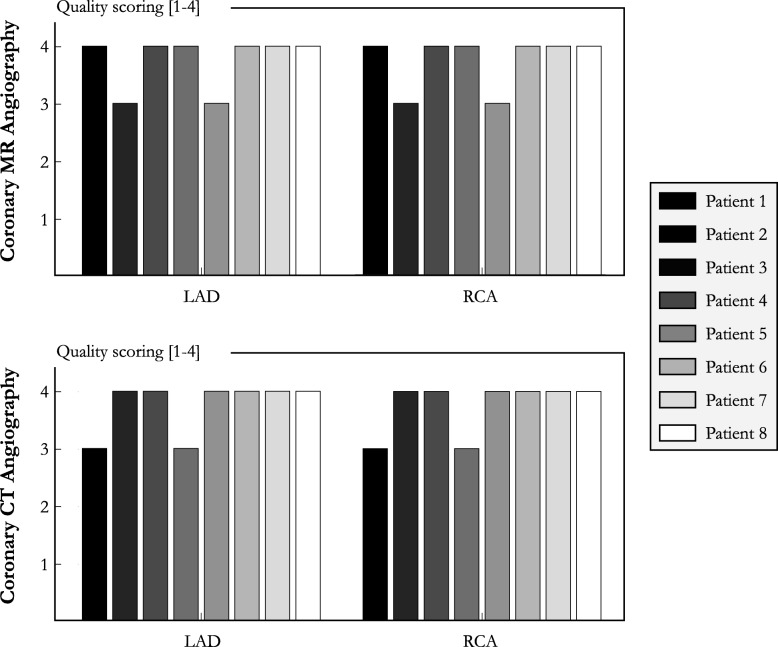
Qualitative coronary results for the patients (*N* = 8) who underwent accelerated free-breathing whole-heart sub-millimeter isotropic CMRA with the proposed non-rigid motion-compensated PROST reconstruction and CCTA are provided for both coronary arteries. A score of 4 indicates excellent image quality with sharply defined coronary borders. Abbreviations: LAD, left anterior descending artery; RCA, right coronary artery

Vessel sharpness of the first 4 cm was in agreement with the healthy subject results (LAD: 54.8 ± 7.7%, RCA: 57.8 ± 6.3%) and no statistical difference was found between LAD and RCA (*P* = 0.336). Good CMRA image quality scores were obtained in the patient study for both the LAD and RCA (Fig. 8, LAD: 3.8 ± 0.5, RCA: 3.8 ± 0.5) similar to CCTA (LAD: 3.8 ± 0.5, *P* = 1.00, RCA: 3.8 ± 0.5, *P* = 1.00), and were comparable to those obtained in the healthy subject study. Additional file 5 shows the image quality improvement achieved in three patients when correcting for non-rigid motion in addition to translational motion correction only.

## Discussion

In this study, we demonstrate the feasibility of combining high undersampling factors with non-rigid motion-compensated PROST reconstruction to obtain high-quality free-breathing 0.9 mm^3^ resolution 3D whole-heart Cartesian CMRA images in a clinically feasible scan time of ~ 8 min. Similar to [22, 29], the proposed reconstruction framework estimates 2D translational motion (FH and LR) from low-resolution iNAVs, which are then used to correct for beat-to-beat respiratory motion. Non-rigid motion-compensated highly-undersampled 3D CMRA images are then obtained using a novel NR-PROST reconstruction which explicitly integrates the estimated inter-bin non-rigid motion in an iterative undersampled 3D patch-based reconstruction framework.

The proposed CMRA acquisition and reconstruction framework was first evaluated in 10 healthy subjects. An acceleration factor of 5x was used to achieve total imaging times of ~ 6 min for 0.9 mm^3^ spatial resolution with 100% respiratory scan efficiency and predictable scan time. The proposed NR-PROST reconstruction was compared to the previously published TC-PROST [29] and non-rigid motion-compensated NR-SENSE [22] reconstruction techniques. Translation correction with undersampled PROST reconstruction enables the visualization of the left and right coronaries with high image quality. However, although sub-millimeter resolution permits the visualization of small structures down to approximately 1 mm, the distal segments of the coronary arteries can be missed in subjects with more complex non-rigid respiratory-induced motion of the heart. Incorporating non-rigid motion correction in NR-SENSE allows for better visualization of the distal segments of the coronary arteries with sharp edges. This advantage, however, has to be balanced with some increase in noise in the reconstructed CMRA image, mainly due to the severely ill-posed reconstruction problem. The proposed NR-PROST approach overcomes this limitation by reconstructing CMRA images with higher image quality (as attested by two clinical experts), with good visualization of distal segments of the coronary arteries and high image sharpness.

The preliminary performance of the proposed CMRA framework in a clinical setting was assessed by imaging 8 patients with suspected coronary artery disease. The proposed approach allowed for the acquisition of the CMRA images in a total acquisition time of ~ 8 min with 100% respiratory scan efficiency. Three of the patients had normal coronary arteries, whereas the remaining five patients had evidence of coronary disease both on non-contrast CMRA and contrast-enhanced CCTA. The proposed non-rigid motion-compensated NR-PROST reconstruction generated high-quality images (with quality scores similar to the healthy subject study) despite high acceleration factors, with good delineation of the coronary vasculature and preservation of local features and luminal narrowing in calcified coronary segments. While the preliminary results on healthy subjects and patients show the potential of the proposed framework, further validation on a larger cohort of patients needs to be performed in future studies. The sensitivity and particularly, the negative predictive values of the presented approach need to be assessed, and its accuracy in detecting coronary stenosis and coronary artery occlusion remains to be evaluated.

In this study, a VD-CASPR sampling was employed with an acceleration factor of 5x to achieve sub 10-min acquisition time with 100% scan efficiency, whereas a fully sampled acquisition would have required clinically impractical scan times (> 40 mins). Besides the use of undersampling, respiratory motion compensation (2D beat-to-beat translation and 3D bin-to-bin non-rigid) also permits a substantial reduction in scan time by allowing 100% scan efficiency and predictable scan time, as opposed to other CMRA techniques based on diaphragmatic-navigator-gated acquisition [3, 4]. For the proposed approach, each of the 5 respiratory bins were reconstructed independently using a fast (i.e., less than 18 s per bin) soft-gating SENSE reconstruction that provides sufficient quality to extract non-rigid motion fields. However, motion-resolved CMRA images with higher image quality could be obtained by jointly reconstructing the bins using recently developed motion-resolved reconstructions, such as XD-GRASP [41] or XD-ORCCA [42], which also exploit sparsity along the respiratory dimension. While those techniques have shown great potential in providing high-quality motion-resolved CMRA images, their computational costs (currently ~ 2 h at a resolution of 1 × 1 × 2 mm^3^ [42]) and memory requirements may not be justified when the objective is solely motion estimation, as in this study.

A first beat-to-beat 2D translational motion correction with iterative SENSE reconstruction was performed inline in the scanner software, to provide a preliminary motion corrected CMRA image that can be visualized immediately after scanning. However, the proposed NR-PROST reconstruction was implemented and performed offline, with its main implementation in Matlab and the multi-threaded 3D patch-based denoising in C, resulting in total reconstruction times of ~ 50 min. Nonetheless, future implementations taking advantage of GPUs and coil compression strategies may significantly reduce the reconstruction time. It should also be emphasized that, compared to other CMRA sequences such as volume-targeted CMRA [2, 43] or diaphragmatic-based navigation [3, 4], the use of 2D iNAVs substantially reduce the operator dependence since only a small 2D box has to be positioned approximately around the heart to track the respiratory motion. Moreover, this 2D box can be defined (or re-defined) off-line during reconstruction. Furthermore, isotropic acquisitions allow whole-heart acquisition without knowledge of the position and course of the coronary arteries, providing almost full independence of the plane of acquisition and therefore avoiding long and tedious planning.

Our data demonstrated that free-breathing whole-heart 0.9 mm^3^ isotropic resolution CMRA permits the evaluation of the coronary arteries over a length of about 12 cm (LAD) and 14 cm (RCA). This is not only important for the non-invasive interrogation of the proximal and mid- segments of the coronary arteries and their collateral branches but, together with curved multiplanar reconstruction, it shows the capacity to localize small coronary lesions and related pathology. While comparison with CCTA showed good agreement and delineation of the coronary arteries with the proposed CMRA framework, CCTA still has the advantage of being less sensitive to motion (faster acquisition, breath-holding) and provides higher spatial resolution (in the order of 0.6 mm^3^). Nonetheless, initial clinical results from this feasibility study demonstrate that free-breathing non-contrast whole-heart sub-millimeter isotropic CMRA using NR-PROST reconstruction holds promise for the safe non-invasive assessment of coronary stenosis without the requirement for intravenous contrast or exposure to ionizing radiation. Future clinical studies comparing the proposed CMRA images with CCTA in a larger cohort of patients under similar pharmacological preparation are needed.

## Conclusions

A framework was developed to enable free-breathing 3D whole-heart sub-millimeter isotropic resolution Cartesian CMRA. The proposed approach achieved fast and predictable acquisition times in healthy subjects and patients (~ 8 min) with good image quality and sharp delineation of the coronary arteries. Ultimately, this non-invasive and radiation-free framework might be a useful tool for rapid screening and comprehensive examination of the coronary vasculature, including the distal segments, in patients with suspected coronary artery disease. Future clinical validation is now warranted.

## Supplementary information

**Additional file 1.** Undersampled 3D variable-density spiral-like Cartesian trajectory (VD-CASPR) to allow for fast acquisition of the high-resolution 3D CMRA data. The Cartesian trajectory with spiral-like order samples the k_y_-k_z_ phase encoding plane following approximate spiral interleaves on the Cartesian grid with variable density along each spiral arm. In this sketch, the 2 first acquired spirals are shown, each spiral containing 20 segments. A golden angle rotation between successive spirals is applied to allow for pseudo-random distribution of the spiral interleaves during respiratory binning.

**Additional file 3.** Example CMRA reconstructions from 3 healthy subjects acquired with the proposed approach with acceleration 5x and 0.9 mm^3^ isotropic resolution are shown. The total acquisition time, with 100% scan efficiency, is expressed as [min:s].

**Additional file 4.** Non-contrast whole-heart sub-millimeter isotropic CMRA images of 3 patients acquired at 0.9 mm^3^ isotropic resolution and reconstructed with the proposed non-rigid PROST framework. The total acquisition time, with 100% scan efficiency, is provided for each patient.

**Additional file 5.** Reformatted CMRA for 3 patients acquired with isotropic resolution of 0.9 mm^3^ and reconstructed with translational PROST (TC-PROST) and the non-rigid PROST framework (NR-PROST). Reformatted sub-millimeter isotropic CMRA along the LAD and RCA for 3 patients with suspected coronary artery disease and reconstructed with translational correction only (TC-PROST, top row) and the proposed non-rigid PROST approach (NR-PROST, bottom row). Accelerated non-rigid PROST allows for improved visualization of both LAD and RCA compared to translation-only corrected PROST. Abbreviations: bpm, beat per minute; CCTA, coronary CT angiography; TC-PROST, translational correction PROST; NR-PROST, non-rigid PROST.

## Data Availability

The datasets used and/or analyzed during the current study are available from the corresponding author on reasonable request.

## Abbreviations

3D: Three dimensional
ADMM: Alternating direction method of multipliers
bSSFP: Balanced steady state free precession
CAD: Coronary artery disease
CCTA: Coronary computed tomography angiography
CMRA: Coronary magnetic resonance angiography
ECG: Electrocardiogram
FA: Flip angle
FH: Foot-head
FOV: Field-of-view
iNAV: Image based navigator
LAD: Left anterior descending coronary artery
LCA: Left coronary artery
LCX: Left circumflex coronary artery
NR: Non-rigid
PDA: Posterior descending coronary artery
PROST: Patch-based low-rank reconstruction
RCA: Right coronary artery
RL: Right-left
SPIR: Spectral presaturation with Inversion recovery
VD-CASPR: Variable density spiral like Cartesian trajectory
