# Supplementary figures and images for: 3D whole-heart isotropic sub-millimeter resolution coronary magnetic resonance angiography with non-rigid motion-compensated PROST
Source: J Cardiovasc Magn Reson. 2020 Apr 16;22:24. doi: 10.1186/s12968-020-00611-5 (PMC7161114; doi:10.1186/s12968-020-00611-5)

# VD-CASPR sampling

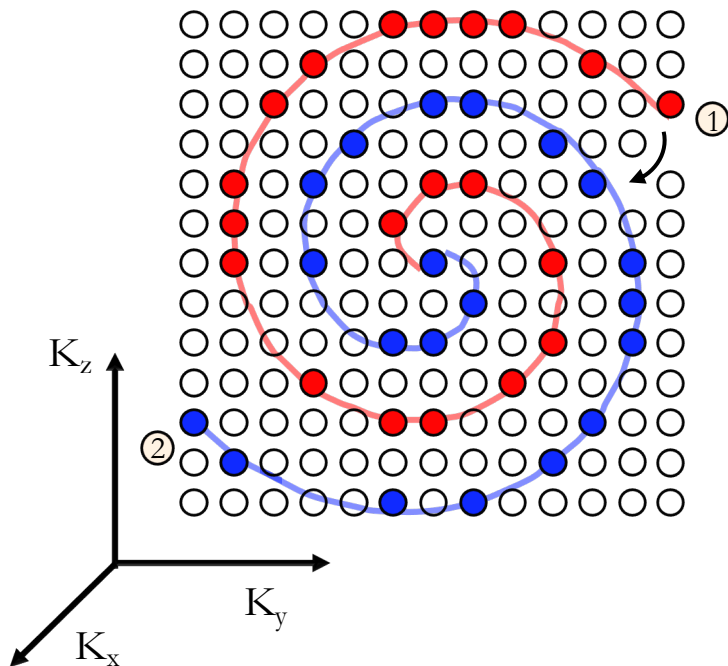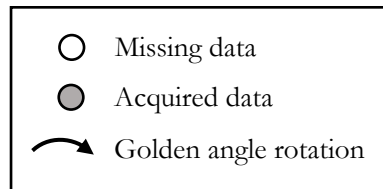

Supplement: Supplementary file 2 — Additional file 1. Undersampled 3D variable-density spiral-like Cartesian trajectory (VD-CASPR) to allow for fast acquisition of the high-resolution 3D CMRA data. The Cartesian trajectory with spiral-like order samples the ky-kz phase encoding plane following approximate spiral interleaves on the Cartesian grid with variable density along each spiral arm. In this sketch, the 2 first acquired spirals are shown, each spiral containing 20 segments. A golden angle rotation between successive spirals is applied to allow for pseudo-random distribution of the spiral interleaves during respiratory binning. [file 12968_2020_611_MOESM1_ESM.pdf]

Patient 7 – 35 yo 57 bpm

Patient 6 – 53 yo 51 bpm

Patient 8 – 54 yo 57 bpm

TC-PROST

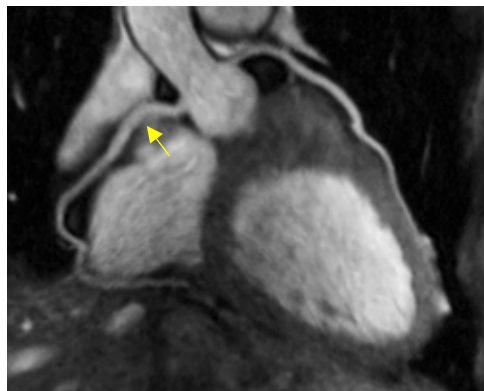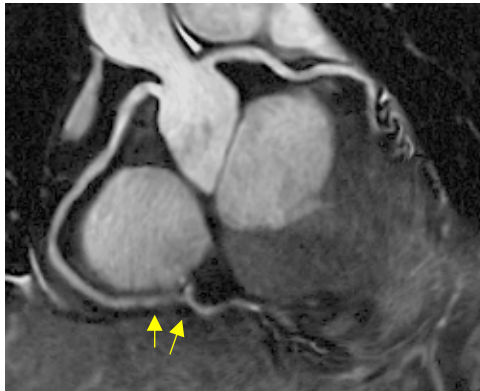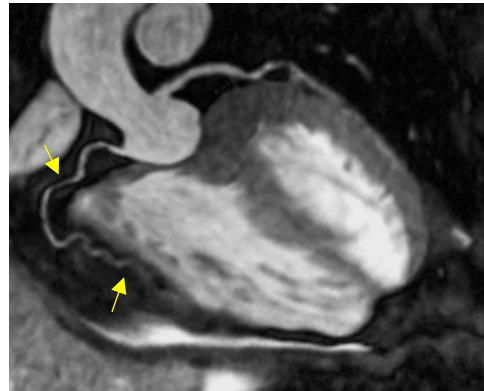

NR-PROST

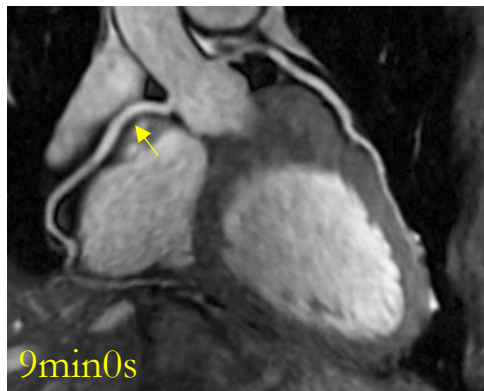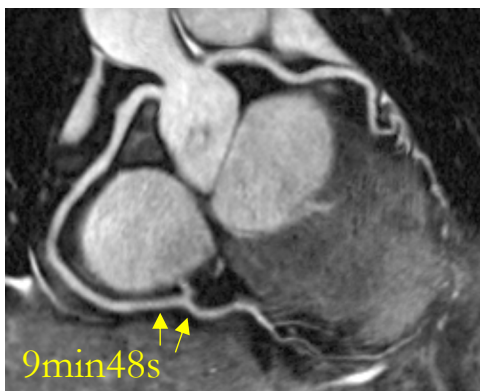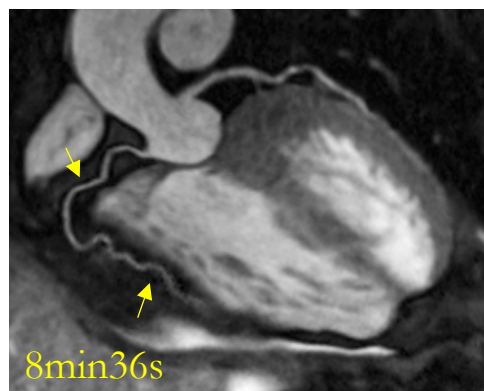

Supplement: Supplementary file 5 — Additional file 5. Reformatted CMRA for 3 patients acquired with isotropic resolution of 0.9 mm3 and reconstructed with translational PROST (TC-PROST) and the non-rigid PROST framework (NR-PROST). Reformatted sub-millimeter isotropic CMRA along the LAD and RCA for 3 patients with suspected coronary artery disease and reconstructed with translational correction only (TC-PROST, top row) and the proposed non-rigid PROST approach (NR-PROST, bottom row). Accelerated non-rigid PROST allows for improved visualization of both LAD and RCA compared to translation-only corrected PROST. Abbreviations: bpm, beat per minute; CCTA, coronary CT angiography; TC-PROST, translational correction PROST; NR-PROST, non-rigid PROST. [file 12968_2020_611_MOESM5_ESM.pdf]
